# Yellow Fever

**Published:** 1878-10-20

**Authors:** T. B. Greenley

**Affiliations:** Ky.


### YELLOW FEVER.

BY T. B. GREENLEY, M. D., OF KY.

There is quite a controversy of late among medical men in regard to the contagiousness, and portability of yellow fever both in the medical journals and secular press of Louisville.

Some of our ablest brothers are arrayed on either side of these important questions; among whom stand Drs. Blackburn, Gaillaird etc., on the affirmative, and Drs. T. S. Bell, L. P. Yandell etc. on the negative, whilst a few take middle ground, and say that it is not personally contagious, but may be transmitted by means of clothing, bedding, &c. At the head of this theory stands Surgeon General Woodworth of the U. S. Marine Hospital service.

Now where such prominent and able men in the profession as these differ so widely in opinions and *theories*, how are such differences to be settled except by facts as developed by the history of the disease?

Dr. Bell strongly takes the ground that yellow fever is the product of malaria, and of course endemic in its origin. He maintains that sixty days of solar heat sufficient to cause a mean range of 76 degrees F. with moisture and vegitable matter in due quantities, will generate malaria strong enough to produce the disease. He also affirms that we never have in the Ohio valley solar temperature sufficiently protracted to engender this plague, and hence it is impossible for for it to become epidemic in Louisville and other cities and towns on the Ohio. According to the history of the disease in Louisville, there never has been a case aside from those who had lately arrived from infected localities.

Dr. Blackburn on the other hand maintains that the disease is both contagious and portable, and that it is exotic in its origin. He is of the opinion that the portable principle consists of a microscopic germ that may be carried in clothing, or even in ballast on board of ships from point to point, and when brought in contact with persons living in localities where a great deal of uncleanliness and filth exists may develop the disease. He also believes that a person in health may take it from a patient sick with the fever. Either of these views or theories would seem to be more reasonable than that of Surgeon General Woodworth. He says in his circular respecting quarantines that a well person will not take the disease from a sick one, but may take it from his clothes. Now this idea at first sight even strikes the mind as palpably unreasonable. It is as much as to say that the poison, or contagion or germ, or microphyte or whatever principle it may be that produces the disease is much stronger after it leaves the body of the patient and enters his clothing or bedding, than it is as it emanates from his body. That idea is too absurd to be entertained for a moment. Were it true the question would arise how shall we treat yellow fever without clothing or bedding? We might tax the genius of some inventive mind to construct a matrass of hair or very fine wire and dispense with clothing. Male and female patients could be attended by nurses of their own sex, and thus stop the spreading of the disease.

On the portability and contagiousness of the disease depends the entire question of the utility or non utility of quarantines. If it is not portable nor contagious then quarantining against it is a great and monstrous outrage, both against the people of infected places and the commerce of the country. It is a question of momentous import, and should demand the attention of government authorities. There should be a commission appointed of the most learned and scientific men of our profession, who are versed in the history of epidemics, and who could set aside preconceived *theories*, and investigate and collect *facts* in regard to the habitues of yellow fever so as to enable them to report unbiased and profitably. If it should be ascertained that it is not contagious and cannot be transported, then the barbarous and inhuman quarantine should be abolished.

Now which of these theories is the most plausible? We will test them by facts. Yellow fever never prevails in cold weather, in fact cold weather abates it at once where it is prevailing. It always selects localities where there is a great deal of filth or in close proximity to marshes or malarious places after long protracted solar heat.

CLEANLINESS AS A SURE PROPHYLACTIC.

If a person goes from an infected locality to one where the disease never has prevailed he may take it, but no one else will take it from him.

This fact is being now fully verified every day in Louisville. Many persons are going there from New Orleans, Memphis and other places in the South where they are taken sick and die, but not a single case has yet originated there.

Some might say that these facts do not prove the disease not to be contagious.

We are not trying to prove a negative, but leave the other side to prove the affirmative—that it is contagious. If it can be shown that a single case occurred independent of contagion or portable germ then that theory falls to the ground.

Doctor Woodhull, Surgeon U. S. A. who went to Savannah to enquire into the cause and study the history of the epidemic of 1876 in that city, after a thorough investigation came to the conclusion that it was of local origin.

He was strongly prejudiced in favor of the importation theory.

His report is published in the July Number of the "American Journal of Medical Sciences" for 1877. He says: "The condition of Savannah in the summer of 1876 may be epitomized as follows: On the west, north, and southeast, and in a less degree on the east there were badly drained lowlands that had been flooded by unusually heavy rains in June; on three sides, but especially on the west, large amounts of garbage were deposited near the city; the sewers gave off foul gases, sufficiently to attract general attention; the supply of the city waterworks were drawn from contaminated sources; the soil of the city was probably saturated with the products of animal decomposition, and the wells from which much of the drinking water was drawn penetrated this soil; to the east at an average distance of 700 yards was the open and practically stagnant sewer, known as the Bilbo canal, and 400 yards further east was a rice plantation in full cultivation.

While as stated at the outset, I began this investigation with the expectation of tracing the epidemic to an imported source. I have been led step by step, contrary to my anticipations to these conclusions, to wit: that it is of local origin and dependent on the unhealthy condition of the city and its surroundings.

The result of Dr. Woodhull's investigation, which was thorough and exhaustive, and the facts of its local origin being so strong and convincing as to change his preconceived theory of the exotic origin of yellow fever should have great weight in the minds of all members of the profession who have not had equal opportunities to study its characteristics. Much credit is due him for his great pains taking investigation and extremely able and unprejudiced report.

I look upon it as offering, not only strong but unanswerable argument in favor of the local origin of the Savannah epidemic of 1876, as well as being dependent upon the presence of malaria in a virulent form.

The germ theorists all agree that certain conditions must exist in the localities where the contagious principle is caused before it can take root and produce the disease. There must be hot weather, filthy streets, etc; in other words there must be malaria in due quantity to furnish the germ a nidus for its proper development.

Now you see that if it was not for this little mite of a germ, this inconceivable, infinitesi mal microphyte, the contagio-portable theorists might shake hands with the malarial theorists.

At first view it would seem that such a small atom might be gotton over without stumbling, but in this it appears we are to be disappointed. That imaginary particle is to be held out. It must be cousin-german to that invisible agent that travels over the world producing cholera.

Laying all theories and prejudices aside I believe all the phenomena of yellow fever can be accounted for by taking malaria as its cause of production. It has its synonim. to some extent, in some of the known malarial troubles; to wit: conjestion, remittent, pernicious intermittent, and if you please cholera. It generally attacks in the night, and is attended with suppression of urine, resembling cholera in these regards. It prevails in localities and at the season of the year and under circumstances when and where we might expect known malarial diseases to prevail, and when all the elements are present to constitute malaria. It is cut short by the very weather that we would expect to oust other malarial diseases. As it resembles known malarial troubles in some of its characteristics, would it not be well to try the great anti-malarial remedy as a prophylactic? Dr. Bell highly recommends the free use of quinine in this way, and as it is an antidote to malaria generally, I have no doubt that if persons living in infected localities would keep their systems saturated with this remedy it would prevent the development of the disease.

Dr. Bell believes that cholera and tropical dysentery, as well as yellow fever depends on malaria for their cause, but in varying degrees of intensity. He also believes in the latency of these affections; that persons may after leaving localities where the diseases are prevailing, have them developed in places even where they could not originate. This has occurred frequently in regard to yellow fever and cholera.

A circumstance occurred recently that might by the casual observer be construed to favor the theory of portability and contagion. I alluded to the case of the steamer John Porter, which left New Orleans in July some time after the yellow fever prevailed in that city and made her way up the Mississippi as far as St. Louis, thence to Louisville and up the Ohio as far as Gallipolis. some distanee above Cincinnati. Most of her crew had either left or died before reaching Louisville, and new hands were obtained. She was quarantined at Gallipolis and not allowed to permit any one to go ashore. Several of the recruits took the disease aboard and died. Now it seems to me that the development of the disease aboard that boat can be very easily accounted for on the malarial theory, without paying any attention to the imaginary germ. All the reporters who went on board of her represent her condition as far as filth, and stench from bilge water were concerned, to be most intolerable. She verily brought the New Orleans poison with her, and as the weather continued hot during her trip several weeks it did not abate any of its virulence. A person confined on board of that boat with all the surroundings were just as much exposed to the cause of yellow fever as he would have been in an infected district in New Orleans.

